# Arseniate of Lithia for Diabetes

**Published:** 1887-09

**Authors:** 


Arseniate of Lithia for Diabetes.—Dr. Martineau recommends carbonate of lithia, 3 grs.; arseniate of soda, 1-10 gr.; carbonic acid water, 2 pints. Solution is effected under pressure. The effervescing liquid is taken mixed with claret, the foregoing dose to last for at least three days, being taken at the two principal meals of the day customary in Paris. No change of diet is necessary. Dujardin - Beaumetz and others are skeptical about the value of this treatment, but it is simple and easy, and when the patient is not dangerously ill, it will do no harm to try it.—*Technics.*

